# Personal and Household Hygiene, Environmental Contamination, and Health in Undergraduate Residence Halls in New York City, 2011

**DOI:** 10.1371/journal.pone.0081460

**Published:** 2013-11-27

**Authors:** Benjamin A. Miko, Bevin Cohen, Katharine Haxall, Laurie Conway, Nicole Kelly, Dianne Stare, Christina Tropiano, Allan Gilman, Samuel L. Seward, Elaine Larson

**Affiliations:** 1 Division of Infectious Diseases, College of Physicians and Surgeons, Columbia University, New York, New York, United States of America; 2 Center for Interdisciplinary Research to Reduce Antimicrobial Resistance, Columbia University, New York, New York, United States of America; 3 Department of Epidemiology, Mailman School of Public Health, Columbia University, New York, New York, United States of America; 4 Columbia University School of Nursing, New York, New York, United States of America; 5 Department of Biology and Medical Laboratory Technology, Bronx Community College, Bronx, New York, United States of America; 6 Columbia University Health Services, New York, New York, United States of America; Universitätsklinikum Hamburg-Eppendorf, Germany

## Abstract

**Background:**

While several studies have documented the importance of hand washing in the university setting, the added role of environmental hygiene remains poorly understood. The purpose of this study was to characterize the personal and environmental hygiene habits of college students, define the determinants of hygiene in this population, and assess the relationship between reported hygiene behaviors, environmental contamination, and health status.

**Methods:**

501 undergraduate students completed a previously validated survey assessing baseline demographics, hygiene habits, determinants of hygiene, and health status. Sixty survey respondents had microbiological samples taken from eight standardized surfaces in their dormitory environment. Bacterial contamination was assessed using standard quantitative bacterial culture techniques. Additional culturing for coagulase-positive *Staphylococcus* and coliforms was performed using selective agar.

**Results:**

While the vast majority of study participants (n = 461, 92%) believed that hand washing was important for infection prevention, there was a large amount of variation in reported personal hygiene practices. More women than men reported consistent hand washing before preparing food (p = .002) and after using the toilet (p = .001). Environmental hygiene showed similar variability although 73.3% (n = 367) of subjects reported dormitory cleaning at least once per month. Contamination of certain surfaces was common, with at least one third of all bookshelves, desks, refrigerator handles, toilet handles, and bathroom door handles positive for >10 CFU of bacteria per 4 cm^2^ area. Coagulase-positive *Staphylococcus* was detected in three participants' rooms (5%) and coliforms were present in six students' rooms (10%). Surface contamination with any bacteria did not vary by frequency of cleaning or frequency of illness (p>.05).

**Conclusions:**

Our results suggest that surface contamination, while prevalent, is unrelated to reported hygiene or health in the university setting. Further research into environmental reservoirs of infectious diseases may delineate whether surface decontamination is an effective target of hygiene interventions in this population.

## Introduction

Hand hygiene has been shown to reduce the incidence of respiratory and gastrointestinal infections [1]. Despite its simple, cost-effective nature, adequate hand washing is rarely practiced, even in developed countries where hygiene supplies are readily available [2], [3], [4]. While all individuals would likely benefit from improved hygiene practices, certain populations may be particularly impacted by directed hygiene interventions. Students in the university setting may be ideal targets given their transition from family to independent living, increased risk for infectious diseases [5], and potential for effective behavioral modification. Shared living spaces, close physical contact, and variable hygiene likely contribute to the enhanced transmission of infectious agents in the dormitory setting [5]. Bacterial contamination of common surfaces in both personal and shared dormitory spaces may contribute additional risk. While environmental contamination has been documented in household settings, its role in transmission of infectious pathogens remains unclear [5]. The aims of this study were to (1) describe the knowledge, practices, and beliefs about personal and environmental hygiene held by college students living in dormitories; (2) examine and quantify the microbial flora present on surfaces in students' dormitory rooms and bathrooms; (3) determine whether there is an association between reported knowledge, practices, and beliefs about personal and home hygiene and frequency of illness; and (4) determine whether there is an association between microbial flora found in dormitory environments and frequency of illness.

## Methods

### Ethics

This research was approved by the Columbia University Medical Center Institutional Review Board (CUMC IRB) and conducted with the assistance of Student Health Services, Housing Services, and Dining Services. All participants were given an information sheet describing the study and provided verbal informed consent. Those completing environmental sampling provided full written informed consent.

### Sample and Setting

This study was conducted among undergraduate students at Columbia University in New York City in the Fall 2011 semester. Participants were recruited at the larger of the University's two dining halls. All Columbia University freshmen are required to purchase a meal plan and therefore utilize the dining hall on a regular basis. Students entering the dining hall were eligible to participate in the study which included (1) completion of a standardized hygiene questionnaire and (2) culturing of environmental dormitory surfaces in a subset of subjects. Recruitment procedures are detailed in our previous research in this population, and briefly described below [6].

### Questionnaire

The study survey included 60 questions of varying styles (Likert-scale, multiple choice, and open-ended answers) and was based on a validated hygiene metric that had been previously piloted by our research team in this setting [6]. The instrument retrospectively assessed specific aspects of health and hygiene over the preceding 30 days. Areas of question included personal hygiene behaviors, household hygiene behaviors, beliefs and knowledge surrounding hygiene, and reported health status. The presence of particular symptoms (e.g., cough, fever, diarrhea), missed classes due to illness, visits to a health care provider, and use of prescription or non-prescription medications were assessed as measures of health status. Several questions on hygiene activities addressed the frequencies of reported behaviors in specific scenarios (e.g., frequency of hand washing before preparing food, after using the toilet, etc.). All individuals participating in the study completed the questionnaire. Participants undergoing environmental sampling had their survey data linked with bacterial culture results.

### Data Collection

Trained research assistants enrolled participants at the dining hall during dinnertimes on various days of the week. Students were approached upon entrance and offered information on study aims and procedures. Those agreeing to complete a questionnaire offered verbal consent prior to survey initiation. Upon completion of the instrument, participants were compensated ten dollars for their time. The environmental sampling protocol was subsequently explained to study participants. Those wishing to volunteer for this portion of the study were asked to provide contact information to schedule the study visit to their living space. Environmental sampling visits occurred within two weeks of survey completion. Two research assistants were present at each residence hall visit and obtained written informed consent for microbiologic sampling, as outlined below. Prior to microbiological sampling, we did not inform prospective participants of the exact visit timing or of the surfaces being assessed. As participants were asked to clean their room with their normal frequency, they were not asked when individual surfaces were last cleaned. These participants were compensated an additional twenty dollars for their time.

### Specimen Collection and Processing

Microbiological samples were collected using pre-moistened rayon-tipped culturette swabs (Becton Dickinson, Franklin Lakes, NJ). A 4 cm^2^ area of the following surfaces was cultured in each participant's living environment: computer keyboard, bookshelf, desk, reusable cup/dish, remote control (television or other device), overhead light switch, refrigerator handle, and bathroom stall/door handle. Refrigerated specimens were transported to the laboratory and processed after an average time of 36 hours (range 12 to 72 hours). Serial dilutions of each specimen in phosphate buffered saline (e.g., undiluted, 1∶10, 1∶100) were inoculated onto sheep's blood agar plates and incubated at 35°C for 24 hours. Colony forming units (CFU) counts were determined using a binocular dissecting microscope. Environmental contamination with coagulase-positive *Staphylococcus* (e.g., *S. aureus*) and coliforms was assessed using direct inoculation onto selective agar (mannitol salt and MacConkey, respectively). No broth enrichment was performed. Probable *Staphylococcus aureus* was further confirmed using the tube coagulase test. Quantitative cultures were not obtained for these organisms.

### Statistical Analysis

Data analysis was conducted using SPSS software (PASW Statistics 18.0; IBM SPSS, Armonk, NY). Survey data were analyzed with Pearson's chi-squared test for categorical variables (e.g., Likert scale responses). Quantitative environmental cultures were analyzed as dichotomous variables as several specimens showed CFUs that were too numerous to count. The >10 CFUs cut point was chosen for this dichotomous analysis because of its clinical relevance as an inoculation dose for particular pathogens (e.g., S. aureus) and its statistical discrimination (>10 CFUs represented the upper quartile of our environmental specimens). As with the survey data, Pearson's chi-squared was used to assess independence of microbiological results. For the survey portion of our study, pre-enrollment sample size analysis demonstrated sufficient power to detect clinically meaningful differences between comparison groups (95% power for odds ratios of 2 or greater for total sample size of 500). Our ability to detect statistical differences in environmental contamination was more limited (80% power for odds ratios of 4 or greater for a total sample size of 60). Statistical significance was set to alpha less than or equal to 0.05. Participants included in our previous study were not included in this analysis.

## Results

A total of 501 students completed the study survey. Subject demographics are listed in Table 1.

**Table 1:** Baseline demographics of study participants (n = 501).

Variable	%(n)^a^
Age	19 years (mean)
Gender	50.1% men (251)
	49.7% women (249)
	0.2% transgender (1)
Academic concentration	43.6% science majors (218)
	37.4% humanities majors (187)
	19% undecided or other (95)
Class year	40.9% freshmen (204)
	30.5% sophomores (152)
	22.4% juniors (112)
	6.2% seniors (31)
Dormitory style	48% hall style (240)
	52% suite style (260)
Number of roommates^b^	42.9% no roommates (215)
	55.9% one roommate (280)
	1.2% two or more (6)

a Frequencies do not match total N for all questions because not all respondents answered every question.

b Subjects residing in suite style housing were more likely to have one or more roommates compared to those living in hall style housing (67.4% v. 48.3%, respectively).

### Reported hygiene habits

Reported hand hygiene practices varied greatly among study participants. Subjects noted a median of 5 hand hygiene events per day (range 0 to 30 times), each lasting 16.5 seconds on average (range 0 to 70 seconds). While the large majority of students (n = 474, 94.6%) reported washing their hands “always” or “most of the time” after using the toilet, hand hygiene was significantly less common in other scenarios: 246 students (49.1%) reported washing their hands “always” or “most of the time” prior to meals; 50% reported that they practiced hand hygiene after touching a pet or other animal. The relationships between reported personal hygiene practices and demographic factors are shown in Table 2. Women were significantly more likely than men to report washing their hands always or most of the time in all scenarios except before eating. Lower classmen were significantly more likely than upper classmen to report washing their hands after using the toilet. Declared major did not significantly predict hand hygiene practices. Liquid hand soap was the most common product used for hand hygiene (n = 418, 83.4%) with a minority regularly using hand sanitizer (n = 179, 35.7%). 5.4% (n = 27) of study subjects perceived hand sanitizer as more effective than soap and water.

**Table 2:** Differences in college students' knowledge, practices, and beliefs about hand hygiene by gender, class standing, and academic major.

	Women N = 249^b^	Men N = 251^b^	*p* a	Lower classmen N = 356^b^	Upper classmen N = 143^b^	*p* a	Science majors N = 218^b^	Humanities/undecided majors N = 282^b^	*p* a
**Knowledge**									
***Hand washing can*** **…**									
*Prevent transmission of cold or flu:*									
Yes	93 (232)	90 (226)	0.207	91 (324)	93 (133)	0.486	92 (201)	91 (257)	0.670
No/don’t know	7 (17)	10 (25)		9 (32)	7 (10)		8 (17)	9 (25)	
*Prevent transmission of gastroenteritis:*									
Yes	88 (220)	85 (214)	0.307	87 (310)	86 (123)	0.751	86 (188)	87 (246)	0.744
No/don’t know	12 (29)	15 (37)		13 (46)	14 (20)		14 (30)	13 (36)	
**Practices**									
***Do you wash your hands*** **…**									
*Upon getting home:*									
Always/most of the time	39 (98)	29 (73)	0.015	34 (122)	34 (48)	0.881	35 (77)	33 (93)	0.583
Sometimes/never	61 (151)	71 (178)		66 (234)	66 (95)		65 (141)	67 (189)	
*Upon finishing a workout at a gym:*									
Always/most of the time	69 (171)	57 (143)	0.008	66 (232)	57 (81)	0.076	65 (142)	61 (171)	0.317
Sometimes/never	31 (77)	43 (106)		34 (122)	43 (61)		35 (75)	39 (109)	
*After touching a pet or other animal:*									
Always/most of the time	60 (149)	40 (100)	<0.001	52 (184)	46 (66)	0.240	51 (111)	49 (138)	0.587
Sometimes/never	40 (100)	60 (149)		48 (170)	54 (77)		49 (105)	51 (144)	
*Before eating:*									
Always/most of the time	53 (133)	45 (113)	0.061	49 (174)	50 (72)	0.766	50 (109)	48 (136)	0.694
Sometimes/never	47 (116)	55 (138)		51 (182)	50 (71)		50 (109)	52 (146)	
*Before preparing food:*									
Always/most of the time	92 (229)	83 (206)	0.002	89 (315)	83 (119)	0.080	86 (187)	88 (247)	0.421
Sometimes/never	8 (20)	17 (43)		11 (39)	17 (24)		14 (31)	12 (33)	
*After using the toilet:*									
Always/most of the time	98 (244)	92 (230)	0.001	96 (342)	91 (130)	0.021	95 (208)	94 (265)	0.480
Sometimes/never	2 (5)	8 (21)		4 (14)	9 (13)		5 (10)	6 (17)	
**Beliefs**									
***Do you think your personal hygiene is*** **…**									
Better than others'	40 (100)	39 (97)	0.925	39 (138)	40 (57)	0.496	41 (90)	38 (107)	0.542
About the same as others'	55 (135)	56 (140)		55 (196)	56 (80)		55 (119)	56 (157)	
Worse than others'	5 (13)	5 (14)		6 (22)	4 (5)		4 (9)	6 (17)	
***For preventing disease, hand washing is*** **…**									
Important	93 (232)	91 (228)	0.336	92 (326)	93 (133)	0.594	95 (208)	89 (252)	0.013
Unimportant/neutral	7 (17)	9 (23)		8 (30)	7 (10)		5 (10)	11 (30)	

a Chi-square test for independence.

b Frequencies do not match total N for all questions because not all respondents answered every question.

Data are percent (n).

Similar to personal hygiene, household hygiene habits varied greatly among study participants. While the majority of subjects reported that their living space was cleaned on a weekly basis (n = 314, 62.7%), a subset reported cleaning on a daily basis (n = 53, 10.6%) or monthly basis (n = 61, 12.2%). 10.8% (n = 54) of study subjects stated that their room was “never” cleaned. Such variability was seen in the individual surfaces sampled. While 273 subjects (54.5%) reported desktop cleaning at least once per month, 345 (68.9%) stated that they cleaned their keyboard less than once per semester. Infrequent cleaning (once per semester or less) was common for many surfaces including bookshelves (n = 358, 71.5%), television remote controls (n = 425, 84.8%), light switches (n = 414, 82.6%), and refrigerator handles (n = 261, 72.7%). Disposable dishes or cups were cleaned frequently (daily n = 280, 55.9%; weekly n = 114, 22.8%). Cleaning of bathroom doors and toilet flush handles was frequent for a subset of study participants (at least once weekly, n = 205, 40.9% and n = 225, 44.9%, respectively). More study participants utilized disinfecting products compared to non-disinfecting products (n = 299, 59.7% v. n = 165, 32.9% respectively). Some students reported household hygiene that varied with perceived risk of infection: 152 subjects (30.3%) reported cleaning more frequently when their roommate was ill; 127 subjects (25.3%) cleaned more frequently when residents of their floor were sick.

### Reported hygiene beliefs

The vast majority of study participants believed that hand washing was important for infection prevention (n = 461, 92%). Most students expressed an understanding that hand hygiene was instrumental in preventing upper respiratory infections (n = 459, 91.6%) and gastrointestinal infections (n = 435, 86.8%). Nearly 80% of study participants (n = 399) reported that disinfection was important for preventing infection. Beliefs and knowledge surrounding hygiene did not vary based on gender or class standing (Table 2); science majors noted that hand washing was important in preventing disease more frequently than non-science majors. Most study participants perceived their hygiene habits to be equal or better than other classmates. Only 5.4% (n = 27) noted that their personal hygiene was worse than other students; 11% (n = 55) reported that their household hygiene was worse than others'. Study subjects noted diverse determinants of their hygiene habits. Family influence was most commonly reported (n = 384, 76.6%), followed by education (n = 331, 66.1%), peers (n = 285, 56.9%), and work experience (n = 277, 55.3%).

### Reported health status

The vast majority of study participants (n = 495, 98.8%) described their health as either “excellent” or “good;” 79.6% (n = 399) reported no medical comorbidities. The most commonly noted health conditions were asthma (n = 45, 9%) and seasonal allergies (n = 55, 11%). Type 1 diabetes mellitus (n = 2, 0.4%) and cardiovascular disease (n = 1, 0.2%) were rarely noted. Several survey respondents reported symptoms of an infectious disease over the preceding month. Common complaints included cough (n = 272, 54.3%), runny nose (n = 357, 71.3%), upset stomach (n = 247, 49.3%), vomiting (n = 83, 16.6%), diarrhea (n = 97, 19.4%), and fever (n = 65, 13%). 65 students (13%) missed class over the previous month due to these complaints; 56 (11.2%) sought medical care. While a minority of students took antibiotics (n = 30, 6%), several took prolonged courses (up to 30 days), corresponding to 167 days of antibiotic use among study participants.

### Microbiologic results

Microbiologic samples of the dormitory environment were collected from 60 study participants (30 men and 30 women). Bacterial contamination of specific surfaces was variable, ranging from no growth to CFUs too numerous to count. Surface contamination showed little variation by type of dormitory, reported frequency of cleaning, or reported frequency of illness among the subset of study participants undergoing environmental sampling (n = 60, Table 3). Coagulase-positive *Staphylococcus* was detected in three participants' rooms (on a dish, bookshelf, and remote control) and coliforms were present in six students' rooms (on a remote control, keyboard, desk, light switch, refrigerator handle, bathroom door handle, and three bookshelves). Two of these students reported cleaning daily, three weekly, two monthly, and one never.

**Table 3:** Microbial flora on dorm room surfaces by type of dorm, frequency of cleaning, and frequency of illness.

	N	Computer keyboard	Bookshelf	Desk	Television remote	Overhead light switch	Reusable cup or dish	Refrigerator handle	Toilet flush handle	Bathroom stall/door handle
**Type of dorm**										
Hall style	30	13 (4)	43 (13)	33 (10)	7 (2)	7 (2)	13 (4)	37 (11)	27 (8)	37 (11)
Suite style	30	7 (2)	70 (21)	33 (10)	17 (5)	0 (0)	17 (5)	40 (12)	43 (13)	33 (10)
*p* a		0.389	0.037	1.0	0.228	0.150	0.718	0.791	0.176	0.787
**Frequency of dorm room cleaning**										
At least once per week	45	11 (5)	53 (24)	27 (12)	16 (7)	4 (2)	13 (6)	38 (17)	31 (14)	31 (14)
Less than once per week	15	7 (1)	67 (10)	53 (8)	0 (0)	0 (0)	20 (3)	40 (6)	47 (7)	47 (7)
*p* a		0.619	0.367	0.058	0.104	0.406	0.531	0.878	0.274	0.274
**Frequency of illness**										
Zero days	14	7 (1)	64 (9)	14 (2)	14 (2)	0 (0)	14 (2)	50 (7)	29 (4)	43 (6)
At least one day	46	11 (5)	54 (25)	39 (18)	11 (5)	4 (2)	15 (7)	34 (16)	37 (17)	33 (15)
*p* a		0.684	0.511	0.084	0.727	0.427	0.932	0.305	0.565	0.581

a Chi-square test for independence.

Data are percent (n) of surfaces with >10 colony forming units.

## Discussion

The college dormitory setting has been recognized as a community-based reservoir for infectious diseases [5]. Similar to military barracks, college dormitories house large numbers of young adults in close proximity, often with variable infection control practices. While *Neisseria meningitides* has received particularly intense study due to its morbidity and mortality, numerous bacterial and viral pathogens spread efficiently and cause disease in this setting [7]. Outbreaks of influenza, non-influenza respiratory viruses, measles, mumps, varicella, and rubella have all been noted among residents of college dormitories [5], [8], [9], [10]. Several studies have demonstrated that improved hygiene behaviors – particularly hand washing – are effective in reducing the incidence of certain infections such as viral upper respiratory infection and gastroenteritis [5], [11]. Although a growing literature has clarified the determinants and effects of particular hygiene behaviors, many practices remain incompletely understood [12].

Little is known about the prevalence or significance of environmental contamination in the university setting. In 2009, Brooke et al. sampled 70 commonly touched surfaces on a university campus for *S. aureus* [13]. Several objects showed a high burden of bacterial contamination, with over 90% of computer keyboards positive when cultured late in the day. While methicillin-susceptible *S. aureus* (MSSA) was isolated from several surfaces (computer keyboards, telephones, and elevator buttons), no methicillin-resistant (MR) strains were found. Two recent studies by Roberts et al. examined *S. aureus* surface contamination at the University of Washington (specifically, the dental school clinic, general university campus, student homes, and surrounding community) [14], [15]. These studies demonstrated a substantial burden of MRSA environmental contamination (4.1% to 8.4% of surfaces) with variable prevalence of the epidemic strain USA300. While our study demonstrated significant bacterial surface contamination, coagulase-positive *Staphylococcus* was infrequently found.

The role of the residential environment has been a focus of particularly controversial debate in recent years. Several studies have implicated household fomites in the transmission of infectious diseases, including viral and bacterial enteric pathogens [16], [17], [18], [19], [20]. In the non-outbreak community setting, case reports have documented recurrent staphylococcal infections that have been resolved only after various household surfaces were decontaminated [21], [22]. Despite such observational data, interventional studies have yielded mixed results. A 2004 randomized controlled trial by Larson et al. noted no reduction in symptoms from viral infectious diseases in households that utilized antimicrobial cleaning and hand washing products [23]. In contrast, a 2008 study by Sandora et al. found that a multifactorial hygiene campaign including decontamination of classroom surfaces reduced the risk of gastroenteritis among a cohort of school children [24]. Taken together, there is sufficient evidence to conclude that several organisms persist and transmit in the inanimate environment. Whether such contamination has clinically meaningful effects on infection risk is unanswered at present.

The inconclusive nature of this literature is likely multifactorial and may reflect the heterogeneity of community-based cohorts. It is possible that in certain populations at elevated risk for infection – including college dormitory residents – environmental contamination may be of increased importance, thereby mimicking healthcare populations rather than lower risk community residents. Despite this hypothesis, our study failed to show an association between contamination of dormitory surfaces and (1) dormitory style, (2) reported household hygiene, and (3) reported illness. Such a finding may reflect that environmental colonization is inconsequential in this setting. As this is the first study of dormitory residents to evaluate the relationship between environmental colonization, reported hygiene habits, and reported health status, it is not possible to assess for congruence with other studies. Our results suggest that surface contamination, while prevalent, is unrelated to hygiene or health in the college setting. If this finding is confirmed, hygiene interventions targeting the environment may be ill suited for this population, despite the perception of their poor cleaning habits.

Several aspects of our study design warrant consideration when interpreting these results. Although >95% of Columbia University undergraduates live in residence halls and participate in the University-sponsored dining plans, upper year students and those with dietary restrictions are less likely to purchase meal plans and may be underrepresented in our sample. In addition, sample size may have limited our ability to identify statistically significant associations. Although the power to detect moderate risk relationships was sufficient for the survey component of the study, the microbiological component was powered only to detect large determinants. Hygiene habits and health status were based on self-reported data and consequently subject to recall and reporting bias. Previous studies have shown that self-reported hygiene surveys often overestimate true hygiene behavior, sometimes substantially [25]. It is likely that any bias present in these self-reports, however, would be over-reporting of hygienic practices. Hence, the relatively clean environment was particularly surprising.

Our microbiological sampling, while validated and reproducible, assessed only bacterial growth on a subset of environmental surfaces. Although the surfaces cultured represented a standardized set of commonly-touch items present across all dormitory settings, other surfaces remained untested. Whether environmental sampling of additional surfaces, particularly those in common or public spaces, would have altered study finding is unknown. As viral illnesses, particularly upper respiratory infections and gastroenteritis, are common in the dormitory setting, bacterial contamination will not reflect the etiology of these diseases. Although a causal link between bacterial colonization and viral infection is implausible, we hypothesize that surface contamination is a marker of poor hygiene, which itself has been linked with viral upper respiratory infections and gastroenteritis. While study subjects were told to continue with baseline cleaning habits prior to environmental culturing, it is possible that participants altered household hygiene prior to our assessment, further impeding our quantification of surface contamination. The presence or absence of clinical infection prior to microbiological sampling may have altered endogenous bacterial shedding into the environment or cleaning habits. While every effort was made to culture surfaces in a timely manner, some subjects were cultured up to two weeks after survey completion, further impairing our comparison of questionnaire data with microbiological findings. Specimens were refrigerated after collection and cultured efficiently. Despite this, processing times showed variability (12 to 72 hours, average 36 hours) and we were not able to assess for differences in isolation or quantification of bacteria between these time points. Taken together, our sampling technique, use of survey data, and sample size may have weakened our ability to quantify the association between household hygiene, environmental contamination, and health.

Despite these limitations, our study provides new insight into the relationship between hygiene and health in the college dormitory setting. While cleanliness itself may be a meaningful marker of safe hygiene practices, environmental contamination appears unrelated to reported household hygiene and risk of clinical infection. Our data suggest that most college students have a clear understanding of hygiene benefits and place significant belief in its ability to prevent infection and promote health. As such, this population may be well suited for hygiene interventions with sustained impact over adult life. Whether household hygiene should be a target of these initiatives remains unclear. Further study into environmental reservoirs of infectious diseases may delineate the importance of surface contamination and define the relative impact of household hygiene interventions in this important setting.
